# Combinatorial optimization of synthetic operons for the microbial production of *p*-coumaryl alcohol with *Escherichia coli*

**DOI:** 10.1186/s12934-015-0274-9

**Published:** 2015-06-11

**Authors:** Philana V van Summeren-Wesenhagen, Raphael Voges, Alexander Dennig, Sascha Sokolowsky, Stephan Noack, Ulrich Schwaneberg, Jan Marienhagen

**Affiliations:** Institut für Bio- und Geowissenschaften, IBG-1: Biotechnologie and Bioeconomy Science Center (BioSC), Forschungszentrum Jülich, 52425 Jülich, Germany; Department of Chemistry, Organic and Bioorganic Chemistry, University of Graz, Heinrichstrasse 28, 8010 Graz, Austria; Lehrstuhl für Biotechnologie and Bioeconomy Science Center (BioSC), RWTH Aachen University, Worringer Weg 1, 52056 Aachen, Germany

**Keywords:** *p*-Coumaryl alcohol, Plant natural products, Metabolic engineering, Synthetic operon, Phosphorothioate, Balanced gene expression

## Abstract

**Background:**

Microbes are extensively engineered to produce compounds of biotechnological or pharmaceutical interest. However, functional integration of synthetic pathways into the respective host cell metabolism and optimization of heterologous gene expression for achieving high product titers is still a challenging task. In this manuscript, we describe the optimization of a tetracistronic operon for the microbial production of the plant-derived phenylpropanoid *p*-coumaryl alcohol in *Escherichia coli*.

**Results:**

Basis for the construction of a *p*-coumaryl alcohol producing strain was the development of Operon-PLICing as method for the rapid combinatorial assembly of synthetic operons. This method is based on the chemical cleavage reaction of phosphorothioate bonds in an iodine/ethanol solution to generate complementary, single-stranded overhangs and subsequent hybridization of multiple DNA-fragments. Furthermore, during the assembly of these DNA-fragments, Operon-PLICing offers the opportunity for balancing gene expression of all pathway genes on the level of translation for maximizing product titers by varying the spacing between the Shine-Dalgarno sequence and START codon. With Operon-PLICing, 81 different clones, each one carrying a different *p*-coumaryl alcohol operon, were individually constructed and screened for *p*-coumaryl alcohol formation within a few days. The absolute product titer of the best five variants ranged from 48 to 52 mg/L *p*-coumaryl alcohol without any further optimization of growth and production conditions.

**Conclusions:**

Operon-PLICing is sequence-independent and thus does not require any specific recognition or target sequences for enzymatic activities since all hybridization sites can be arbitrarily selected. In fact, after PCR-amplification, no endonucleases or ligases, frequently used in other methods, are needed. The modularity, simplicity and robustness of Operon-PLICing would be perfectly suited for an automation of cloning in the microtiter plate format.

**Electronic supplementary material:**

The online version of this article (doi:10.1186/s12934-015-0274-9) contains supplementary material, which is available to authorized users.

## Background

Tools and methods of synthetic biology have become increasingly important to design, assemble and optimize biosynthetic pathways for the biotechnological production of fine chemicals [1], biofuels [2], pharmaceuticals [3] or natural products [4].

Tuning of gene expression from such synthetic pathways can be achieved on the level of transcription or translation. Transcription in particular has been in the focus of metabolic engineering since many years, as a myriad of different (inducible) natural promoters have been successfully used for the tuning of gene expression. Furthermore, engineering of the promoter architecture has achieved tunable gene expression from synthetic promoters in various industrially relevant platform organisms such as *Escherichia coli* [5] or *Saccharomyces cerevisiae* [6]. Gene expression on the translational level can be controlled via adaptation of the codon usage during the design of the synthetic genes [7], modulation of the mRNA-stability [8], application of metabolite-responsive riboswitches [9] or design of the ribosomal binding site (RBS) [10]. However, considering the complexity of gene expression, a balanced expression of multigene pathways for achieving optimal catalytic efficiencies still represents a major challenge, especially if the genes originate from different organisms. Combinatorial assembly and subsequent evaluation of many different pathway variants can be a very time consuming and expensive task depending on the cloning method employed. Many DNA assembly techniques have been developed over the past few years to complement the more traditional cloning, which is based on the use of enzymes for in vitro DNA restriction and ligation [11]. Among the more prominent methods for the assembly of pathways are polymerase cycling assembly (PCA) [12], Gibson assembly [13], the sequence and ligation independent cloning (SLIC) method [14] or USER-fusion [15].

The phosphorothioate-based ligase-independent gene cloning (PLICing) method has been developed as an enzyme-free and sequence-independent cloning method of single genes [16]. PLICing starts with amplification of the target gene and the vector by PCR using primers with complementary phosphorothioated nucleotides at the 5′-end. The PCR products are cleaved in an iodine/ethanol solution, yielding single-stranded overhangs. Subsequently, these ends hybridize at room temperature and the resulting DNA constructs can be directly transformed into competent host cells. PLICing is sequence independent and has found numerous applications in the field of protein engineering for performing simultaneous site-saturation of five codons in single genes [17, 18] and for recombining secondary structure elements, motifs or domains of single proteins [19].

In this study, we developed Operon-PLICing for the rapid assembly of synthetic pathways and included the possibility to simultaneously tune (“balance”) the expression of all pathway genes on the level of translation. This balancing allows for maximizing product titers as well as for minimizing formation of inclusion bodies and accumulation of potentially cytotoxic pathways intermediates.

## Results and discussion

**Figure 1:**
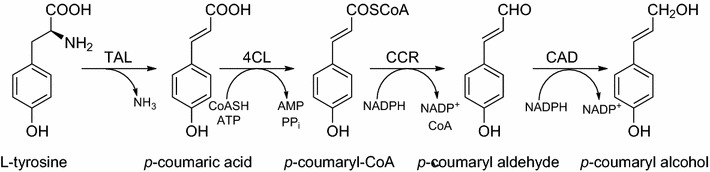
Biosynthetic pathway for the production of *p*-coumaryl alcohol from l-tyrosine.

Model pathway for the development of this method was a four-step, hybrid plant/bacterial pathway to convert the amino acid l-tyrosine to the monolignol *p*-coumaryl alcohol (Figure 1). This plant natural product is an important precursor of pharmaceutically interesting lignans and key building block of the plant polymer lignin. *p*-Coumaryl alcohol synthesis from l-tyrosine starts with a deamination step catalyzed by a tyrosine ammonia lyase (TAL, EC 4.3.1.23) to form *p*-coumaric acid. Subsequently, this acid is coenzyme A (CoA)-activated by a 4-coumarate-CoA-ligase (4CL, EC 6.2.1.12). The resulting *p*-coumaryl-CoA is reduced to *p*-coumaryl alcohol in two subsequent steps by a cinnamoyl-CoA-reductase (CCR, 1.2.1.44) and a cinnamyl alcohol dehydrogenase (CAD, EC 1.1.1.195). The heterologous production of *p*-coumaryl alcohol was recently established in *Escherichia coli*, and without precursor feeding a final product titer of 22 mg/L *p*-coumaryl alcohol could be achieved [20]. The low titer was explained by the observed cytoplasmatic aggregation of insoluble heterologous proteins in inclusion bodies. At the beginning of our experiments, four genes encoding the tyrosine ammonia lyase from *Rhodobacter sphaeroides* (RsTAL), the 4-coumarate-CoA-ligase from *Petroselinum crispum* (Pc4CL), as well as cinnamoyl-CoA-reductase (ZmCCR) and cinnamyl alcohol dehydrogenase from *Zea mays* (ZmCAD) were codon optimized and synthesized for the heterologous expression in *E. coli*. Initially, three plasmid-based variants of a tetracistronic *p*-coumaryl alcohol pathway were constructed. In all cases the transcription was controlled by a single IPTG-inducible T7 promoter and the translation of each gene in the respective pathway was modulated by an individual, but always identical Shine-Dalgarno (SD) sequence (5′-TAAGGAGGT-3′) as prokaryotic ribosome binding site (pALXtreme-1a_T7_prom_>*Rstal*-*Pc4* *cl*-*Zmccr*-*Zmcad*). The pathways differed only in the length (spacing) of the nucleotide sequence between SD sequence and the START codon of each gene. The length of this short spacing is known to have major influence on the translation initiation efficiency [21]. A spacing of 5 nucleotides (nt) between these two regulatory elements confers the optimal translational efficiency, whereas a deviation from this length (either a reduction or an elongation) results in a stepwise decreased translation efficiency. In the first pathway variant (5-5-5-5 variant) this spacing was set to 5 nt for each gene, in the second pathway (9-9-9-9 variant) to 9 nt and in the third pathway (13-13-13-13 variant) to 13 nt with the aim to generate three reference strains with a high, medium and low expression of the four synthetic *p*-coumaryl-alcohol pathway genes.

**Figure 2:**
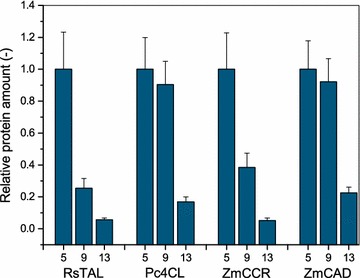
Relative cytoplasmatic protein concentrations of RsTAL, Pc4CL, ZmCCR and ZmCAD when heterologously expressed in *E. coli* BL21 (DE3) *lacI* ^*Q1*^. For each protein, three gene variants with different SD sequence–START codon spacings (5, 9, or 13 nt) were expressed. Relative protein concentrations were determined by IDMS–LC–MS/MS, and results for each protein, whose gene had a 5 nt SD sequence–START codon spacing upstream of the open reading frame were used as reference for comparison.

Subsequently, relative cytoplasmatic protein concentrations in cell free extracts (CFEs) were determined to verify that the variation of the spacing between the SD sequence and the START codon can indeed modulate the translation efficiency for each heterologously expressed protein. This was achieved by targeted proteomics applying isotope dilution mass spectrometry coupled to high performance liquid chromatography (IDMS–LC–MS/MS) [22]. This method using ^15^N-metabolic labeling of the target proteins as internal standards allows reliable peptide and protein quantification in CFEs as complex biological samples. For this purpose, four proteotypic peptides, generated by trypsin-digestion of CFEs, were identified by LC–MS/MS for each enzyme to serve as signature peptides during relative protein quantifications (see Additional file 1: Table S1; Additional file 1: Figure S1). The peptide measurements obtained for the SD sequence–START codon spacing of 5 nt were used as reference for the determination of relative protein amounts for the different gene variants (Figure 2; Additional file 1: Table S2). As expected, the highest cytoplasmatic protein concentrations were obtained with a spacing of 5 nt for all four heterologously expressed genes. With an increasing length of spacing, the relative cytoplasmic amount of all four proteins decreased stepwise. Thereby, each protein showed an individual expression profile. For instance, RsTAL expression was reduced by 75% when increasing the SD sequence–START codon spacing from 5 to 9 nt, while the amount of the Pc4CL was only reduced by 10%.

All three strains were subsequently characterized regarding their capability to produce *p*-coumaryl alcohol to find out if the translation modification is also reflected in product titers. The obtained results showed indeed, that engineered variations of SD sequence–START codon spacing modulate overall product titers: The 5-5-5-5 variant accumulated up to 40 mg/L *p*-coumaryl alcohol, whereas a product concentration of 12 mg/L could be determined in the supernatant of the 9-9-9-9 variant. For the 13-13-13-13 variant the lowest *p*-coumaryl alcohol titer of only 5 mg/L could be measured.

**Figure 3:**
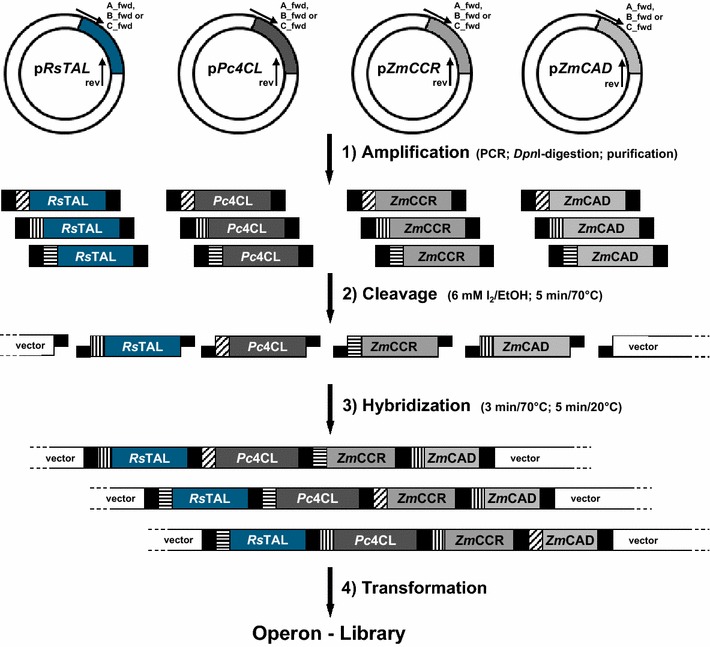
Combinatorial assembly of synthetic *p*-coumaryl-alcohol operons employing Operon-PLICing. Three different variants of each gene, having either a 5, 9 or 13 nt SD sequence–START codon spacing (indicated by *different hachures upstream* of the respective open reading frames) were generated by PCR with phosphorothioate-oligonucleotides. Subsequently, the phosphorothioate bonds were cleaved for generating complementary 3′-overhangs. The resulting twelve different gene fragments and the vector fragment were mixed in 81 independent hybridization reactions to yield all possible combinations of the tetracistronic *p*-coumaryl-alcohol operon prior to transformation to *E. coli.*

**Figure 4:**
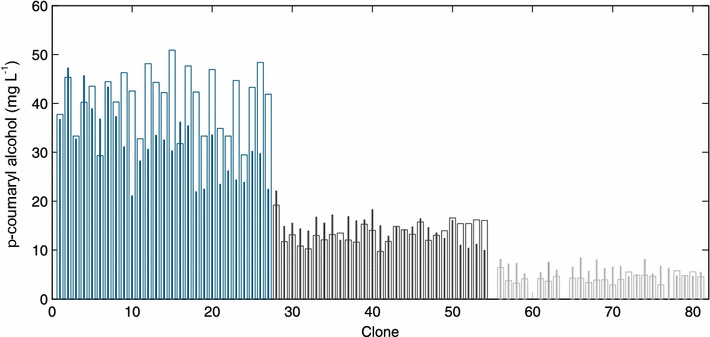
*p*-Coumaryl alcohol titers of all 81 constructed variants of the synthetic *p*-coumaryl alcohol operon. The clones are numbered (and colored) from 1 to 81 in accordance to Table 1. The *blue*, *black* and *grey bars* represent the SD sequence–START codon spacing of the plasmid-encoded *rstal*-gene, which was set to 5, 9 or 13 nt, respectively (*blue* 5 nt; *black* 9 nt; *grey* 13 nt). The microtiter plate screening was performed in duplicate and both product titers for each clone are displayed (first screening: *broad white bar*; second screening: *thin colored bar* *inside the white bar*).

Encouraged by these results we constructed the first combinatorial *p*-coumaryl-alcohol operon library with a variation of the translation efficiency for each gene in the four-step pathway following the Operon-PLICing principle (Figure 3). For this purpose, three versions of each gene, having either a 5, 9 or 13 nt SD sequence–START codon spacing were generated by PCR with phosphorothioate-oligonucleotides (Additional file 1: Table S3). The 12 resulting PCR products and the pALXtreme backbone, which was also PCR amplified with phosphorothioate-oligonucleotides were individually subjected to chemical cleavage of the phosphorothioate bonds for generating complementary 3′-overhangs. Key to the combinatorial assembly of the pathway variants was the design of the complementary sequences required for hybridization of the five individual fragments, which make up a full operon. In this experiment, each of the required five sequences was unique to keep the gene order (*Rstal*-*Pc4cl*-*Zmccr*-*Zmcad*) of the synthetic *p*-coumaryl-alcohol operon during the process of assembly constant. This limited the number of pathway variants to 3^4^ different operon configurations as three different SD sequence–START codon spacings for each of the four genes are possible. Simplicity and robustness of the Operon-PLICing procedure allowed the individual construction of all 81 variants from PCRs to the final library in a few days (Table 1). The completeness of each operon was verified by colony PCR and only a few operon-variants had to be reconstructed as a gene was missing. Subsequently, cultivation and screening in the 96-well plate format for the production of *p*-coumaryl alcohol of this operon library was performed. HPLC-analysis of the supernatant of all strains revealed that the 81 variants can be divided in three distinct groups according to SD sequence–START codon spacing for the gene encoding the RsTAL (Figure 4). This first enzymatic step of the pathway appears to be crucial for the overall pathway performance. All 27 variants (variants 1–27) with a 5 nt spacing upstream of the *Rstal* START codon are characterized by higher product concentrations (up to 50 mg/L *p*-coumaryl alcohol) compared to the 27 variants with a 9 nt spacing upstream of the *Rstal*-gene (variants 28–54; accumulation of up to 24 mg/L *p*-coumaryl alcohol). Only up to 9 mg/L *p*-coumaryl alcohol could be detected in the supernatant of clones 55–81, all harboring operon variants in which the *Rstal*-gene is characterized by a SD sequence–START codon spacing of 13 nt. These results are in accordance with determined kinetic parameters of various plant derived l-tyrosine ammonia lyases as these enzymes generally display a low catalytic activity [23–25]. This would explain the need for maximizing the translation efficiency in the genetic environment of the synthetic *p*-coumaryl alcohol operon to compensate for low TAL activity in *E. coli*. Under microtiter plate screening conditions no general trend for the translation efficiency of the other three enzymes was observable. However, during screening, a few variants within the best performing group accumulated more product compared to the 5-5-5-5 variant, indicating that best translation efficiency for all four pathways genes might not be optimal for achieving highest product titers.

**Table 1:** 81 Synthetic *p*-coumaryl alcohol operon variants differing in the combination of the SD sequence–START codon spacings (5, 9, 13 nt)

Variant	SD seq.–START spacing	Variant	SD seq.–START spacing	Variant	SD seq.–START spacing
1	5-5-5-5	28	9-5-5-5	55	13-5-5-5
2	5-5-5-9	29	9-5-5-9	56	13-5-5-9
3	5-5-5-13	30	9-5-5-13	57	13-5-5-13
4	5-5-9-5	31	9-5-9-5	58	13-5-9-5
5	5-5-9-9	32	9-5-9-9	59	13-5-9-9
6	5-5-9-13	33	9-5-9-13	60	13-5-9-13
7	5-5-13-5	34	9-5-13-5	61	13-5-13-5
8	5-5-13-9	35	9-5-13-9	62	13-5-13-9
9	5-5-13-13	36	9-5-13-13	63	13-5-13-13
10	5-9-5-5	37	9-9-5-5	64	13-9-5-5
11	5-9-5-9	38	9-9-5-9	65	13-9-5-9
12	5-9-5-13	39	9-9-5-13	66	13-9-5-13
13	5-9-9-5	40	9-9-9-5	67	13-9-9-5
14	5-9-9-9	41	9-9-9-9	68	13-9-9-9
15	5-9-9-13	42	9-9-9-13	69	13-9-9-13
16	5-9-13-5	43	9-9-13-5	70	13-9-13-5
17	5-9-13-9	44	9-9-13-9	71	13-9-13-9
18	5-9-13-13	45	9-9-13-13	72	13-9-13-13
19	5-13-5-5	46	9-13-5-5	73	13-13-5-5
20	5-13-5-9	47	9-13-5-9	74	13-13-5-9
21	5-13-5-13	48	9-13-5-13	75	13-13-5-13
22	5-13-9-5	49	9-13-9-5	76	13-13-9-5
23	5-13-9-9	50	9-13-9-9	77	13-13-9-9
24	5-13-9-13	51	9-13-9-13	78	13-13-9-13
25	5-13-13-5	52	9-13-13-5	79	13-13-13-5
26	5-13-13-9	53	9-13-13-9	80	13-13-13-9
27	5-13-13-13	54	9-13-13-13	81	13-13-13-13

All variants of the operon were cloned into the pALXtreme vector backbone [16] (general scheme: pALXtreme-1a_T7_prom_>x-*Rstal*-x-*Pc4cl*-x-*Zmccr*-x-*Zmcad*) and subsequently transformed to *E. coli* BL21 (DE3) *lacI* ^*Q1*^ [relevant characteristics of the genotype: F^−^ *omp*T *hsd*SB(rB^−^, mB^−^) *gal dcm* (DE3)].

For a deeper insight, shake flask experiments for all 27 clones within this group were performed. Since only negligible growth differences could be detected during cultivation, the determined *p*-coumaryl alcohol concentrations were put in relation to biomass. This was done by simply dividing the determined product titers by the optical density (OD_600_) of the respective culture at the point of measurement. Again, no pattern identifying optimal translation conditions for Pc4CL, ZmCCR and ZmCAD could be identified (Additional file 1: Figure S2). However, variant 5-5-5-5 was among the poorest producers, accumulating only up to 10.4 mg/L/OD_600_*p*-coumaryl alcohol. In contrast, the top five producers accumulated 14.6–16.1 mg/L/OD_600_*p*-coumaryl alcohol in the supernatant after 12 h [variants: no. 12 (5-9-5-13); no. 14 (5-9-9-9); no. 15 (5-9-9-13); no. 18 (5-9-13-13); no. 23 (5-13-9-9)]. This supports the notion that maximum translation efficiency for all four enzymes of this pathway has a negative effect on product formation. The decreased translation efficiency of the other enzymes possibly decreases the metabolic burden for the microbial host with respect to protein synthesis, thus balancing the whole heterologous pathway. The absolute product titer of the best five variants ranged from 47.6 to 52 mg/L *p*-coumaryl alcohol without any further optimization of growth and production conditions. Thus, balancing of the synthetic pathway already more than doubled the overall product titer compared to the previously engineered *E. coli* strain expressing the same genes, as this strain accumulated only up to 22 mg/L *p*-coumaryl alcohol without precursor feeding [20].

The modularity and simplicity of the Operon-PLICing method also provides the opportunity to generate the whole operon library of 81 variants in a single reaction tube by simply combining the 12 amplified and cleaved gene fragments and the equally treated plasmid fragment prior to hybridization and transformation of *E. coli*. Such a library was generated and screened within 2 days. However, DNA sequencing of 20 randomly selected clones prior to screening revealed that a considerable fraction of 25% of all clones constructed by this “one-pot”-protocol harbored plasmids with an incomplete *p*-coumaryl alcohol operon. Similarly, an incomplete pathway assembly was also found when employing other methods such as the CLIVA method [26]. Here the number of correctly assembled operon variants already dropped dramatically when more than only three fragments were to be assembled. Nonetheless, 300 clones of the constructed *p*-coumaryl alcohol library were screened for product formation in 96 deep-well plates, using three-times oversampling to statistically ensure screening of 95% of all possible variants [27]. The three individually constructed reference variants (5-5-5-5, 9-9-9-9 and 13-13-13-13) and a strain harboring the empty vector served as controls. Screening, re-screening and sequencing of the best clones successfully identified the 5-9-9-9 variant, which was also among the best variants in the manually generated library. Hence, although generating a considerable fraction of incomplete operons, this fast “one-pot”-protocol for library generation might be favored when limited handling during library generation is desired and when high-throughput screens, e.g. employing FACS, are available.

## Conclusions

In summary, the method for combinatorial assembly of synthetic pathways described in this manuscript allows rapid construction of a large number of operon variants. Operon-PLICing is sequence-independent and thus does not require any specific recognition or target sequences for enzymatic activities since all hybridization sites can be arbitrarily selected. In fact, after PCR-amplification, no enzymes such as endonucleases or ligases, which are frequently used in other methods, are needed. The modularity, simplicity and robustness of Operon-PLICing would be perfectly suited for an automation of cloning in the microtiter plate format. During the combinatorial optimization of the *p*-coumaryl alcohol operon, only the SD sequence–START codon spacing was modulated. However, any other regulatory elements of gene expression could also be varied during pathway balancing. Furthermore, five unique hybridization sequences, limiting the overall complexity of the generated library to only 81 variants, were used in this study. Decreasing the number of these unique sequences but keeping the number of hybridization sites constant, possibly even using only a single sequence, would allow randomization of the gene order and randomization of the number of genes in constructed operon libraries. The latter effect would offer the opportunity to vary the “dosis” of individual genes in the pathway. This would also represent a possible solution to overcome the low TAL activity within the *p*-coumaryl alcohol pathway, which currently limits the product titers in *E. coli*. However, one has to bear in mind that the transformation efficiency drastically decreases with an increasing plasmid size. Currently, downside of this method is the observed low cloning efficiency during library generation in a single reaction tube. Again, the modularity of Operon-PLICing could be a solution. When a prescreening of a first operon library identifies a certain configuration as most beneficial, this parameter can be fixed by simply using other PCR-primer combinations and amplification of different DNA-fragments prior to construction of a subsequent library. In case of the *p*-coumaryl alcohol operon, the 5 nt-*Rstal* fragment, which was identified as being essential for high product titers, can be fused to the vector fragment. This would reduce the overall number of fragments to be randomly assembled during the construction of the next library of synthetic operons with Operon-PLICing.

## Methods

### Strains and plasmids

*Escherichia coli* BL21-Gold (DE3) *lacI*^*Q1*^ and the plasmid pALXtreme-1a were used for cloning and expression purposes [16]. The cells were routinely cultivated on a rotary shaker (170 rpm) at 37°C in Luria–Bertani (LB) medium or on LB plates [LB medium with 1.5% (wt/vol) agar] [28]. If appropriate, kanamycin (50 µg/mL) was added. Growth was determined by measuring the optical density at 600 nm (OD_600_).

### Genes and primers

Genes encoding the tyrosine ammonia lyase from *Rhodobacter sphaeroides* (RsTAL) [GenBank: ABA81174.1], the 4-coumarate: CoA ligase from *Petroselinum crispum* (Pc4CL) [GenBank: X13324.1], the cinnamoyl-CoA reductase from *Zea mays* (ZmCCR) [GenBank: Y15069.1] and the cinnamyl-alcohol dehydrogenase, also from *Z. mays* (ZmCAD) [GenBank: Y13733.1] were chemically synthesized by GeneArt Gene Synthesis services from Life Technologies, a Thermo Fisher Scientific company (Waltham, MA, USA). Genes were codon optimized for expression in *E. coli* employing the proprietary GeneOptimizer software. Oligonucleotide primers were purchased from Eurofins Genomics (Ebersberg, Germany). Primers used in this study are summarized in Additional file 1: Table S3.

### PCR amplification of target genes and vector for Operon-PLICing

All genes and the pALXtreme-1a vector backbone were amplified by PCR from plasmid template DNA. PCRs were performed in a final volume of 50 µL in a T3000 thermocycler from Biometra (Göttingen, Germany) using thin walled PCR tubes (Bio-Budget, Krefeld, Germany). Typically, PCR reactions were composed of 1× KOD Hot Start DNA polymerase buffer (Merck Millipore, Billerica, MA, USA), 1.5 mM MgSO_4_, 0.2 mM dNTP mix, 0.3 µM phosphorothioate forward and reverse oligonucleotides (PTOs) (Additional file 1: Table S3), 20 ng template DNA, and 1 U KOD Hot Start DNA polymerase. PCR-cycling started with an initial denaturation step at 96°C for 2 min. Subsequently, three cycles (96°C, 20 s; 50°C, 30 s; and 72°C, 120 s), followed by 28 cycles (96°C, 20 s; 55°C, 30 s; and 72°C, 120 s) and one fill-up cycle (72°C, 5 min) were performed. All PCR reactions were subjected to *Dpn*I digestion (10 U, 16 h, 37°C) prior to purification of the PCR products using a PCR purification kit (Macherey–Nagel, Düren, Germany) according to manufacturer’s instructions. Subsequently, PCR products were quantified using a NanoDrop ND-1000 spectrophotometer (NanoDrop Technologies, Wilmington, DE, USA). For increased Operon-PLICing efficiency, a second PCR reaction with the same composition was performed using the amplified DNA fragments from the first PCR reaction as template. For this purpose, 30 PCR cycles (96°C, 20 s; 55°C, 20 s; and 72°C, 60 s) and one fill-up cycle (72°C, 5 min) after the initial denaturation step (96°C, 2 min) were performed. Again, the PCR products were purified using a PCR purification kit (Macherey–Nagel, Düren, Germany). Agarose–TAE gel electrophoresis was performed according to standard protocols to confirm the presence and correct size of the amplified genes and the pALXtreme-1a vector backbone [28].

### Construction of the *p*-coumaryl alcohol operon variants using Operon-PLICing

The iodine cleavage reactions for Operon-PLICing were prepared by mixing 4 µL of the PCR amplified genes (concentrations varied from 0.26 to 0.36 pmol/µL) or 1 µL of the PCR amplified vector backbone DNA, which was adjusted to 4 µL with Milli-Q water (0.06 pmol/µL), with 1 µL cleavage buffer (0.5 M Tris–HCl, pH 9.0), 0.6 µL iodine stock solution (100 mM iodine in 99% ethanol), and 0.4 µL Milli-Q water to a final volume of 6 µl in thin-walled PCR tubes. DNA cleavage was performed by incubating the reaction mixtures at 70°C for 10 min (Biometra T3000 thermocycler). The resulting DNA fragments were used directly for hybridization of the DNA fragments without any further purification. DNA hybridization was achieved by sequentially combining the pALXtreme-1a vector backbone, *Rstal*, *Pc4cl*, *Zmccr* and *Zmcad* iodine cleavage mixtures with 5 min incubation intervals at room temperature after the addition of each DNA fragment. After a final incubation step at room temperature for 5 min, 4 µL of this hybridization mix was used to transform 100 µL chemically competent *E. coli* BL21-Gold (DE3) lacI^Q1^ cells by the RbCl method [29].

In order to verify that all four genes of the synthetic *p*-coumaryl alcohol pathway were successfully incorporated into the vector backbone, recombinant clones were analyzed by colony PCR. A master mix with 1× Dream Taq green master mix (Thermo Scientific, Waltham, MA, USA) and 0.3 µM forward and reverse primer (Additional file 1: Table S3) was prepared and 20 µL aliquots of this master mix were dispensed into thin walled PCR tubes. Colonies were directly transferred from LB agar plates into the PCR tubes with sterile toothpicks. Cycling started with an initial denaturation at 95°C for 3 min, followed by 30 cycles (95°C, 30 s; 60°C, 30 s; and 72°C, 4 min) and one fill-up cycle (72°C, 5 min). The PCR reactions were analyzed on agarose–TAE gels. The *p*-coumaryl alcohol operon of randomly selected clones was sequenced to confirm the correct assembly of the synthetic operons.

### Microtiter plate screening and shake flask cultivations for the microbial production of *p*-coumaryl alcohol

Clones of the generated Operon-PLICing-libraries were picked from the LB plates into 96 well microtiter plates (BRAND, Wertheim, Germany) with 100 µL LB medium per well. These precultures were incubated overnight for 16 h at 37°C in a microtron shaker from Infors [900 rpm and 75% humidity, (Bottmingen, Switzerland)]. 96-well deepwell plates (Eppendorf, Hamburg, Germany) with 600 µL LB medium and 1 mM isopropyl-β-d-thiogalactopyranoside (IPTG) per well were inoculated with 6 µL from each preculture. The deepwell plates were incubated at 25°C for 24 h in the microtron shaker (900 rpm, 75% humidity). The cell-free culture medium was harvested by centrifugation (4°C, 3,500*g*, 30 min,) for the determination of product and intermediates. For *p*-coumaryl alcohol production at shake flask scale, 50 mL LB-medium in 500 mL baffled shake flasks was inoculated with 500 µL of an over-night culture and incubated at 37°C and 120 rpm shaking until the OD_600_ reached 0.2. Subsequently, the cultivation temperature was decreased to 25°C and heterologous gene expression was induced with 1 mM IPTG when the OD_600_ reached 0.6. The cultivation continued at 25°C for an additional 24 h. Samples were taken at regular time intervals for product analysis.

### Determination *p*-coumaric acid and *p*-coumaryl alcohol by uHPLC

*p*-Coumaryl alcohol and *p*-coumaric acid concentrations in the cell-free culture supernatant were determined by ultra-high-performance LC (uHPLC). For this purpose, 2 µL of the supernatant was injected into an Agilent 1290 infinity LC (Santa Clara, CA, USA) using 0.1% (v/v) acetic acid in water (buffer A) and 0.1% (v/v) acetic acid in acetonitrile (buffer B) as the mobile phases at a flow rate of 0.4 mL/min. The LC separation was carried out using a ZORBAX Eclipse AAA (3.5 µm, 4.6 × 75 mm) column with a guard cartridge (4.6 × 12.5 mm) from Agilent at 40°C. For an efficient separation, a gradient was used, where buffer B was increased from 15 to 60% over 10 min. Under these conditions, the retention time of *p*-coumaryl alcohol was 4.9 min, whereas *p*-coumaric acid eluted after 5.3 min. The compounds were detected by monitoring the absorption at 275 nm. The *p*-coumaric acid standard was purchased from Sigma-Aldrich (St. Louis, MO, USA), while *p*-coumaryl alcohol was purchased from MicroCombiChem (Wiesbaden, Germany).

### Relative protein quantification

Relative protein quantification was performed by IDMS–LC–MS/MS as previously described [22]. For the generation of ^15^N-labelled signature peptides of each target enzyme to serve as internal standards during analyses, cells were cultivated in M9 defined medium in a lab-scale bioreactor supplemented with 50 µM FeSO_4_ and 1 µM ZnSO_4_. Glucose (4%) was added as carbon source and ^15^NH_4_Cl served as sole nitrogen source.

For the generation of protein samples, shake flask cultures were harvested 6 h after induction with IPTG by centrifugation (4°C, 6,000*g* for 10 min). The cell pellets were washed with a 0.9 wt% NaCl solution and centrifuged again (4°C, 6,000*g* for 10 min). The obtained cells were concentrated 50× in 1 mL lysis buffer [50 mM potassium phosphate buffer, pH 8.0, 2 mM ethylenediaminetetraacetic acid (EDTA), 2 mM dithiothreitol (DTT), supplemented with complete protease inhibitor cocktail (1 697 498, Roche Applied Science)] and shortly incubated on ice. Subsequently, cells were disrupted by sonication for 4 min at 4°C using a Branson sonifier 250 [intensity 2; duty cycle 20%; Branson, (Danbury, CT, USA)]. Cell free extracts (CFE) were harvested by centrifugation (4°C, 16,000*g* for 30 min). Up to 100 µg of sample protein extract and isotopically labeled standard were digested with trypsin and quantified in hybrid triple quad mass spectrometer. For detailed information regarding the MS analysis the reader is referred to the supplementary information (see Additional file 1).

## Additional file

** Additional file 1.** Supplementary Information.

## Abbreviations

SD: Shine-Dalgarno (sequence)
PLICing: phosphorothioate-based ligase-independent gene cloning
TAL: tyrosine ammonia lyase
4CL: 4-coumarate-CoA-ligase
CCR: cinnamoyl-CoA-reductase
CAD: cinnamyl alcohol dehydrogenase
RsTAL: tyrosine ammonia lyase from *Rhodobacter sphaeroides*
Pc4CL: 4-coumarate-CoA-ligase from *Petroselinum crispum*
ZmCCR: cinnamoyl-CoA-reductase from *Zea mays*
ZmCAD: cinnamyl alcohol dehydrogenase from *Zea mays*
