# Supplementary material for: Combinatorial optimization of synthetic operons for the microbial production of p-coumaryl alcohol with Escherichia coli
Source: Microb Cell Fact. 2015 Jun 11;14:79. doi: 10.1186/s12934-015-0274-9 (PMC4464236; doi:10.1186/s12934-015-0274-9)

Supplementary Information

Combinatorial optimization of synthetic operons for the microbial production of *p*-coumaryl alcohol with *Escherichia coli*

Philana V. van Summeren-Wesenhagen[a,b], Raphael Voges[a], Alexander Dennig[c], Sascha Sokolowsky[a,b], Stephan Noack[a], Ulrich Schwaneberg[b,d] and Jan Marienhagen[a,b]*

[a] *Institut für Bio- und Geowissenschaften, IBG-1: Biotechnologie, Forschungszentrum Jülich, D-52425 Jülich, Germany*

[b]*Bioeconomy Science Center, Forschungszentrum Jülich, D-52425 Jülich, Germany*

[c] *Department of Chemistry, Organic and Bioorganic Chemistry, University of Graz, Heinrichstrasse 28, 8010 Graz, Austria*

[d] *Lehrstuhl für Biotechnologie, RWTH Aachen University, Worringer Weg 1, D-52056 Aachen, Germany*

* corresponding author

Table S1 Proteins, peptides, mass transitions and measurement parameters for *p*-coumaryl-alcohol biosynthetic enzymes expressed in *E. coli*. Crude extracts were digested with trypsin and peptides were detected using a 4000QTRAP LC-MS/MS system.

Protein	Parent ion m/z [Da]	Measured product ions (m/z [Da], CE [V])	Sequence	Charge [-]	DP [V]
RsTAL	418.8	y6 (624.4, 19.6), y5 (511.3, 19.6), y4 (440.3, 19.6)	IVLAPPAR	2	61.6
	314.7	y5 (515.3, 13.7), y4 (458.3, 13.7), y3 (387.3, 13.7)	LGAVIR	2	54.1
	844.5	y12 (1076.5, 43.9), y10 (948.5, 43.9), y9 (861.4, 43.9)	LVSIAQGASGASEGTIAR	2	92.7
	854.5	y11 (1140.6, 44.4), y6 (626.4, 44.4), y3 (359.2, 44.4)	LIDLLNSELAPAVPSR	2	93.4
ZmCAD	529.3	y8 (842.5, 25.9), y7 (785.4, 25.9), y5 (559.3, 25.9)	NTGPEDVVVK	2	69.7
	549.8	y9 (961.5, 27.1), y8 (814.5, 27.1), y6 (644.4, 27.1)	HFGLTTPGLR	2	71.2
	676.4	y11 (1011.5, 34.3), y9 (841.4, 34.3), y6 (628.3, 34.3)	GGILGLGGVGHMGVK	2	80.4
	591.3	y9 (1050.5, 29.4), y7 (830.4, 29.4), y6 (731.4, 29.4)	MGYVNEALER	2	74.2
ZmCCR	391.2	y6 (618.3, 18.0), y5 (505.2, 18.0), y4 (390.2, 18.0)	YLDGSAR	2	59.6
	727.4	y8 (949.5, 37.2), y7 (850.4, 37.2), y6 (722.4, 37.2)	TFANAVQAYVDVR	2	84.1
	367.7	y5 (635.3, 16.7), y4 (488.2, 16.7), y3 (359.2, 16.7)	VFESPR	2	57.9
	594.8	y8 (928.5, 29.6), y6 (702.4, 29.6), y5 (539.3, 29.6)	LFPEYPVPAR	2	74.5
Pc4CL	942.5	y12 (1323.7, 49.5), y11 (1252.7, 49.5), y10 (1138.6, 49.5)	GAISTMANPFFTSAEVIK	2	99.8
	516.3	y9 (831.4, 25.2), y8 (700.4, 25.2), y7 (613.4, 25.2)	TVMSGAAPLGK	2	68.8
	656.3	y10 (1099.5, 33.1), y9 (984.5, 33.1), y7 (758.4, 33.1)	IVDPETNASLPR	2	79.0
	576.3	y8 (931.4, 28.6), y7 (818.4, 28.6), y5 (589.3, 28.6)	GYLNDPESTR	2	73.1

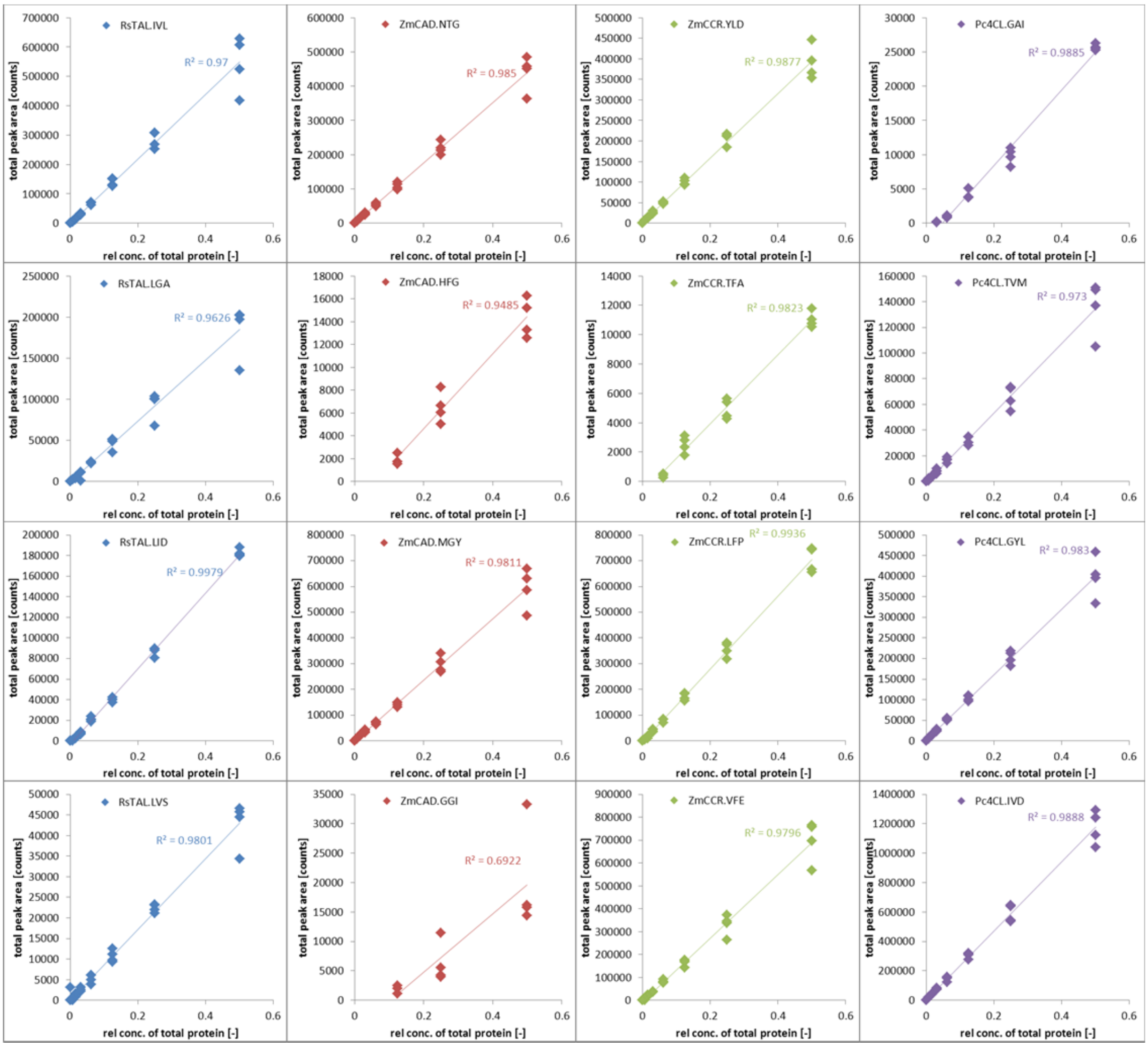

Figure S1 Validation of signature peptides for quantitative measurements. Digested crude extract was diluted sequentially with A. dest and peptide peak areas were measured in the resulting solutions. All 16 signature peptides showed a linear response of peak areas towards the dilution factor.

Table S2 Results of relative quantification of signature peptides from the four enzymes of *p*-coumaryl-alcohol biosynthesis. The peptides ZmCAD.HFG and ZmCCR.TFA could not be used for relative protein quantification (displayed in italics). In case of the first one, signal intensities were to low resulting in inconsistent results, whereas the latter peptide showed retention time instabilities causing peptide elution outside of its measurement window in several samples.

Peptide	5nt	9nt	13nt
RsTAL.IVL	1.00 ± 0.12	0.25 ± 0.03	0.06 ± 0.01
RsTAL.LGA	1.00 ± 0.10	0.27 ± 0.03	0.07 ± 0.01
RsTAL.LVS	1.00 ± 0.13	0.25 ± 0.03	0.06 ± 0.01
RsTAL.LID	1.00 ± 0.12	0.24 ± 0.03	0.05 ± 0.01
ZmCAD.NTG	1.00 ± 0.11	0.91 ± 0.08	0.20 ± 0.02
ZmCAD.HFG	*1.00 ± 0.29*	*1.29 ± 0.53*	*1.75 ± 0.75*
ZmCAD.GGI	1.00 ± 0.10	0.88 ± 0.09	0.21 ± 0.02
ZmCAD.MGY	1.00 ± 0.10	0.97 ± 0.08	0.26 ± 0.02
ZmCCR.YLD	1.00 ± 0.12	0.38 ± 0.04	0.05 ± 0.01
ZmCCR.TFA	*1.00 ± 0.52*	*0.42 ± 0.17*	*0.06 ± 0.03*
ZmCCR.VFE	1.00 ± 0.10	0.39 ± 0.07	0.05 ± 0.01
ZmCCR.LFP	1.00 ± 0.17	0.39 ± 0.05	0.05 ± 0.01
Pc4CL.GAI	1.00 ± 0.13	0.87 ± 0.08	0.15 ± 0.02
Pc4CL.TVM	1.00 ± 0.10	0.89 ± 0.08	0.16 ± 0.02
Pc4CL.IVD	1.00 ± 0.08	0.92 ± 0.07	0.18 ± 0.01
Pc4CL.GYL	1.00 ± 0.08	0.94 ± 0.07	0.18 ± 0.01

Table S3 Phosphorothioate oligonucleotides used in this study. The oligonucleotides were used for the construction of the *p*-coumaryl alcohol operon libraries and oligonucleotides for the verification of the correct operon assembly via PCR.

Primer	Sequence[a] (5´-3´)
Phosphorothioate oligonucelotides (PTOs)	
Vec_PTO_fw	AGCaACTAaTAAgCTAAcAAAgCCCGaAAGgAAGCtGAG
Vec_PTO_rv	ACCtCCTtCTTaAAGTTAAACAAAATTATTTCTAGAGGGGAATTG
RsTAL_5_fw	TAAgAAGgAGGtATACCATGCTGGCTATGAGTCCTC
RsTAL_9_fw	TAAgAAGgAGGtGATCATACCATGCTGGCTATGAGTCCTC
RsTAL_13_fw	TAAgAAGgAGGtATCGGATCATACCATGCTGGCTATGAG
RsTAL_uni_rv	CCAcTTAtCCGgATGATTACTAGTGGGTTATTAAACTGGACTCTGTTGC
Pc4CL_5_fw	CCGgATAaGTGgAATAAGGAGGTATACCATGGGAGATTGTGTAGCAC
Pc4CL_9_fw	CCGgATAaGTGgAATAAGGAGGTGATCATACCATGGGAGATTGTGTAGC
Pc4CL_13_fw	CCGgATAaGTGgAATAAGGAGGTATCGGATCATACCATGGGAGATTGTG
Pc4CL_uni_rv	GAAcAGTgGATcATGATTACTAGTGGGTTATTATTTGGGAAGATCACCG
ZmCCR_5_fw	GATcCACtGTTcAATAAGGAGGTATACCATGACCGTCGTCGACGCCG
ZmCCR_9_fw	GATcCACtGTTcAATAAGGAGGTGATCATACCATGACCGTCGTCGACGC
ZmCCR_13_fw	GATcCACtGTTcAATAAGGAGGTATCGGATCATACCATGACCGTCGTC
ZmCCR_uni_rv	CTGaAGCaCTAgATGATTACTAGTGGGTTATTAGGCACGGATGGCG
ZmCAD_5_fw	CTAgTGCtTCAgAATAAGGAGGTATACCATGGGGAGCCTGGCGTCCG
ZmCAD_9_fw	CTAgTGCtTCAgAATAAGGAGGTGATCATACCATGGGGAGCCTGGC
ZmCAD_13_fw	CTAgTGCtTCAgAATAAGGAGGTATCGGATCATACCATGGGGAGCCTG
ZmCAD_uni_rv	AGCtTCCTtTCGgGCTTtGTTaGCTTaTTAgTTGCtGGCCGCATCCGC
Colony PCR primers	
RsTAL_end_fw	GTTCAGGCACTGCGCGAACAGTTTC
ZmCAD_start_rv	ACCAACAACTTTACGTTCGGACGCCAG

[a]Lower case letters mark the locations of phosphorothioate bases.

Figure S2 Biomass specific *p*-coumaryl alcohol titers of selected *E. coli* clones expressing the *p*-coumaryl alcohol operon variants 1 – 27. Each variant encodes the *Rstal* gene with a SD sequence – START codon spacing of 5 nt. Samples for the determination of *p*-coumaryl alcohol and biomass (OD_{600}) were taken after 12 h of cultivation. The microtiter plate screening was performed in duplicate and both product titers for each clone are displayed (first screening: broad white bar; second screening: thin blue bar inside the white bar).

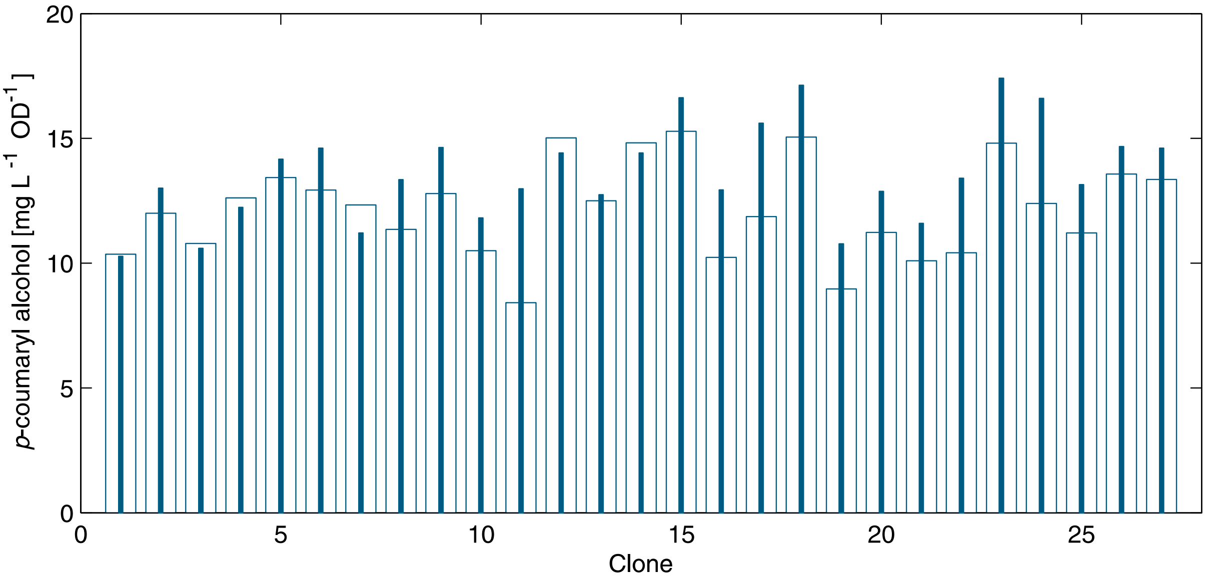

Supplement: Supplementary file 1 — Additional file 1. Supplementary Information. [file 12934_2015_274_MOESM1_ESM.pdf]
